# Indomethacin has no effect on trigeminally provoked parasympathetic output

**DOI:** 10.1177/03331024211030901

**Published:** 2021-08-18

**Authors:** Maike Möller, Celina Schröder, Stefanie Iwersen-Bergmann, Jan Mehnert, Arne May

**Affiliations:** 1 Department of Systems Neuroscience, University Medical Center Eppendorf, Hamburg, Germany; 2 Department of Legal Medicine, University Medical Center Hamburg-Eppendorf, Hamburg, Germany

**Keywords:** NSAID, hemicrania continua, paroxysmal hemicrania, headache, lacrimation

## Abstract

**Background:**

Unlike other non-steroidal anti-inflammatory drugs, indomethacin has been shown to be highly effective in two forms of trigeminal autonomic cephalalgias, hemicrania continua and paroxysmal hemicrania and in some forms of idiopathic stabbing headaches. This specificity is unique in the headache field. Previous findings suggest the involvement of the trigeminal autonomic reflex to play an important role in the pathophysiology of these diseases.

**Methods:**

22 healthy participants were enrolled in a double-blind, three-day within-subject design. The participants received indomethacin, ibuprofen or placebo in a randomized order. After an incubation period of 65 min the baseline lacrimation and the lacrimation during intranasal stimulation evoked by kinetic oscillation stimulation were assessed using Schirmer II lacrimation tests. The lacrimation difference in mm was calculated and compared in a repeated measures ANOVA.

**Results:**

No significant differences were found between the three conditions.

**Conclusion:**

In our study, neither indomethacin nor ibuprofen had an inhibitory effect on the trigeminal autonomic reflex. We suggest that blocking this reflex may not be the treatment mechanism of indomethacin.

## Introduction

The indole acetic acid derivative indomethacin as well as ibuprofen and naproxen belong to the group of the non-steroidal anti-inflammatory drugs (NSAIDs). They act as “pain killers” by non-selectively inhibiting the activity of cyclooxygenase enzymes and thereby inhibiting the synthesis of prostaglandins (1). Although differences in anti-inflammatory activity between the different types of NSAIDs are small, it is striking that indomethacin shows a high specificity in two primary headache disorders, hemicrania continua (HC) and paroxysmal hemicrania (PH) (2). This treatment effect is so unique that both are even defined by their absolute response to indomethacin. Interestingly, other NSAIDs have nearly no effect on these types of headache, forming a sharp contrast to migraine and most other headache diseases (3). Together with cluster headache and the syndrome of short-lasting unilateral neuralgiform headache (SUNCT and SUNA), HC and PH form the group of so-called trigeminal autonomic cephalalgias (TACs) (4). Of note, indomethacin has been shown to have no effect on other TACs. The headache attacks in TACs are characteristically accompanied by cranial autonomic symptoms, such as lacrimation, rhinorrhea, miosis, ptosis, conjunctival injection and facial sweating (4), involving the trigeminal autonomic reflex (5–7). The function of the reflex itself is quite well understood (8) and its activation results in a parasympathetic output, such as lacrimation, elicited by a trigeminal input (6). Several studies suggest that the trigeminal autonomic reflex plays an essential role in the pathophysiology of TACs and also in the therapeutic effect of several treatment approaches (9–12).

Indomethacin inhibits cyclooxygenase activity and therefore reduces the production of prostaglandins (13). Prostaglandins in turn induce the release of calcitonin gene related peptide (CGRP) and nitric oxide (NO) (14–16). Both, CGRP and NO (17,18) are potent vasodilators, able to trigger headache attacks (19–23). Interestingly, several studies have shown that indomethacin reduces the NO induced vasodilation in dural meningeal vessels (24,25). A study by Akerman, using a rodent TAC model has shown that indomethacin reduces firing in the trigeminal cervical complex elicited by superior salivatory nucleus- and also dural-evoked stimulations. This effect was significantly stronger compared to naproxen, another NSAID. Furthermore, the study showed an inhibition of lacrimal duct flow by indomethacin, which was also not affected by naproxen (26), suggesting a specific inhibitory effect of indomethacin on the parasympathetic output. Since naproxen did not show an inhibitory effect, the results of the study may also suggest why indomethacin has this unique therapeutic effect on PH and HC but not, for example, on migraine. However, these studies were performed in rodents and it is not clear whether these effects can be translated into humans.

The aim of the present study was therefore, to investigate the modulatory effect of indomethacin and ibuprofen on the trigeminal autonomic reflex in humans focusing on the possible inhibitory effect of the two medications on the parasympathetic arc of the trigeminal autonomic reflex using the method of nasal kinetic oscillation stimulation (KOS) (5–7).

## Methods

We recruited 25 healthy participants via local internet platforms for the measurement of lacrimation, elicited by kinetic oscillation stimulation (KOS). Exclusion criteria comprised primary headache or any other chronic pain condition, smoking, psychiatric or neurological diseases, contraindications against NSAID or nasal kinetic oscillation, and analgesics intake 24 h before the study. Occasional and rare unspecific headaches following a trauma or an infection were allowed (see Table 1).

**Table 1.** Characteristics of the participants. Age, sex and (unspecific) headache days (per year) of the 22 healthy participants included into the study.

Subject	Age	Sex	Headache days
1	27	f	3
2	31	m	0
3	34	f	2
4	31	m	0
5	19	m	3
6	24	m	1
8	20	f	1
9	28	m	2
10	20	m	3
11	28	m	2
12	23	m	1
13	23	f	3
15	23	f	2
16	18	f	2
17	28	m	0
20	22	f	5
21	23	f	5
22	19	f	3
24	18	m	3
25	21	f	0
27	25	f	2
28	24	f	2
22	24,05	12f ; 10m	2,05

**Figure 1:**
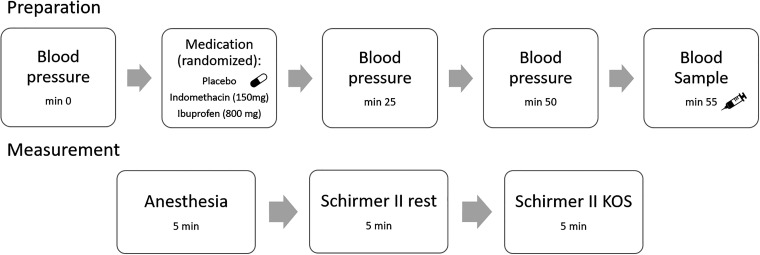
Schematic overview of the study. The blood pressure was assessed prior to the oral medication intake of placebo, indomethacin or ibuprofen in a randomized order. Subsequently, during incubation the blood pressure was assessed 25 min and 50 min later. Blood samples were drawn 55 min after medication intake and the participants were taken to the laboratory for the Schirmer II lacrimation test. For local anaesthesia 1 drop of Conjuncain was administered into each eye and incubated over a time period of five min. Subsequently the Schirmer II lacrimation test at rest and during KOS were performed.

The study included a double-blind, randomized, three-day within-subject design. Participants were instructed not to eat within 2 h before the respective study appointment and were asked to fill in a headache questionnaire, the general health questionnaire German version (PHQ-D) and gave written and informed consent, according to the ethics committee (Ärztekammer Hamburg PV5873) and the Declaration of Helsinki. The participants were asked to lie down in supine position for blood pressure assessment. Subsequently the participants received oral medication, blinded as two identically looking red capsules, containing either ibuprofen (800 mg) indomethacin (150 mg) or placebo. For the incubation period of 65 min, the participants were carefully observed and blood pressure was assessed every 25 min (blood pressure cuff and stethoscope), for a detailed overview see Figure 1. The incubation period of 65 min was chosen, since indomethacin reaches the maximal serum concentration after 30-120 min (27) (Indomet-ratiopharm) and ibuprofen reaches the maximal plasma concentration after 60-120 min (Ibu-ratiopharm). After 55 min a blood sample was drawn (S-Monovette, Sarstedt, Germany) and centrifuged for 10 min at 2500 rpm (Eppendorf, Germany). The blood samples were drawn in order to analyze the serum concentration of the two medications (indomethacin and ibuprofen), respectively. Subsequent to the centrifugation 2 ml serum were taken from the sample and frozen (-25 C°). The analysis of the serum concentration was performed by the department of forensic medicine at the University Medical Center Hamburg-Eppendorf

*Trigeminal autonomic stimulation using KOS procedure.* Kinetic oscillation stimulation (KOS) (Chordate Medical AB, Stockholm, Sweden) is used to treat chronic rhinitis (28). KOS results in a robust and quantifiable cranial autonomic response (5,29,30) For KOS procedure a paraffin-coated single-use catheter was inserted into the left nostril. KOS stimulation consisted of a 6-minute stimulation with a mean pressure of 50 mbar and 40 Hz frequency following previous protocols (5,29,30).

### Measurement of lacrimation

Subsequent to the blood sampling, the participants received local anesthesia, 1 drop per eye (Conjuncain EDO, Bausch+Lomb, Germany) incubated for 5 min. Sterile Schirmer II tear strips (Avimed, Germany) were placed into the lower eye lid for the measurement of lacrimation at rest over 5 minutes. After the measurement at rest, a balloon catheter was moistened with paraffin and placed into the participants’ left nostril. The catheter was attached to a fixation frame placed on the head of the participants, in order to prevent the catheter from moving during the procedure. After an initial tearing reaction, the KOS device was switched on and reached 50 mbar and 40 Hz after an initial ramp up phase. The Schirmer ll strips were placed into the lower eye lid 1 min after start of the KOS and the lacrimation was measured again for 5 minutes. For every minute the lacrimation in mm was filled into a standardized documentation form during measuring at rest and during the KOS procedure.

### Assay for indomethacin and ibuprofen blood levels

An aliquot of a human serum sample (250 µL) was mixed with 50 µL of the internal standard mefenamic acid (0.2 mg/mL). 500 µL acetonitrile was added to precipitate serum proteins. After centrifugation at 14500 rpm for 5 min at 20°C, 100 µL of the supernatant was diluted to a total of 1000 µL with water in a glass microvial. The injection volume was 50 µL.

A high-performance liquid chromatography with diode array detection (HPLC–DAD) method was used for the analysis of ibuprofen and indomethacin serum samples. Chromatography was performed on a Thermo Scientific Accela Liquid chromatography system consisting of an autosampler, quaternary pump and a photo diode array detector with a thermostated column compartment (Thermo Fisher Scientific, Waltham, MA, USA). The chromatographic separation of ibuprofen, indomethacin and internal standard was carried out on a reversed phase Varian Polaris C18-A (250 × 4.0 mm) with particle size of 5 µm and a Varian Polaris® C18-A (250 × 4.0 mm) with particle size of 5 µm and a C18-pre-column (SecuritiyGuard™ Phenomenex®; Aschaffenburg, Germany) using a flow rate of 1.0 mL/min at 25°C. The mobile phase consisted of 37.5% buffer pH 2.3 (consisting of water, 0.66% potassium dihydrogen phosphate, 0.48% ortho-phosphoric acid) and 62.5% acetonitrile. The wavelength detection was set at 276 nm (indomethacin), 219 nm (ibuprofen), and 351 nm (mefenamic acid). Chromeleon™ 7 Chromatography Data Systems Software (Thermo Fisher Scientific, Waltham, MA, USA) was used for the control of the instruments and data acquisition. The assay was calibrated using six standards of spiked blank human serum (2.5, 6.25, 10, 17.5, 25, 32 mg/L for ibuprofen) and (0.3, 1, 1.6, 2.8, 4.0, 5.2 mg/L for indomethacin) there were also two independently prepared quality control samples included in each analytical series. Validation parameters including accuracy, interferences, linearity of calibration and in-process stability complied with international standards. The limit of quantification was 2.5 mg/L for ibuprofen and 0.3 mg/L for indomethacin.

### Assessment of pain

During the measurement of lacrimation, elicited by KOS, the perception inside the nostril was assessed on a visual analogue scale (VAS) for pain between 0 (no pain) and 10 (worst imaginable pain) two minutes after start of the KOS Schirmer II test for each of the three conditions.

### Statistical analyses

For the statistical analysis, three subjects had to be excluded due to more than five unspecific headache days within three months before the study, leaving a sample of 22 participants (12 f; 10 m, age mean±SD=24.05±4.5. One participant experienced adverse events after intake of medication. The acquisition of lacrimation for the respective study day was not possible. During data acquisition at rest for another participant the Schirmer tear strip slipped out of the eye, leading to a missing data set for the respective study day.

For the statistical analysis the lacrimation difference between the KOS-induced lacrimation and the lacrimation at rest in mm for each individual was calculated for each of the three conditions (indomethacin, ibuprofen, placebo). The Schirmer ll tear strips have a maximum capacity of 35 mm. If the maximum was reached, the subsequent time points within the 5 minutes of the Schirmer ll test were labelled as missing values (6,31). Normal distribution for lacrimation, pain and blood pressure were confirmed by a Shapiro-Wilk test and the results were compared in a repeated measures ANOVA. A p-value of <0.05 was considered significant.

## Results

### Measurement of lacrimation

**Figure 2:**
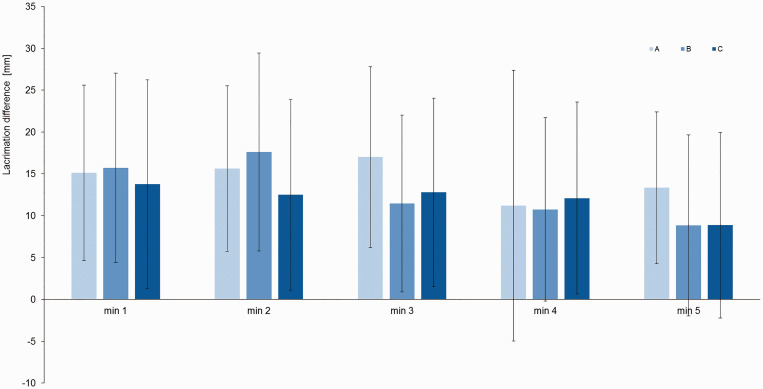
Lacrimation difference between KOS induced lacrimation on the left side and rest after medication intake (n=22). No significant difference was found between the lacrimation difference after Indomethacin (A), Ibuprofen (B) and Placebo (C) over the five minutes of the Schirmer II lacrimation test. The bars represent the mean lacrimation difference and error bars indicate the standard deviation.

No significant difference was observed between indomethacin, ibuprofen and placebo in the calculated repeated measures ANOVA (see Figure 2) for the five minutes of the Schirmer II lacrimation test (minute one mean±SD: A=10.9±11.2; B=9.4±10.8; C=13.9±12.7). Since the assessment of serum level revealed lower medication uptake in nine participants, we repeated the analysis in the 11 participants with high medication uptake (5 f; 6 m, age mean±SD=23.6±5.0), this did not change the results.

### Measurement of blood pressure

No significant difference in blood pressure in the repeated measures ANOVA was found between indomethacin, placebo and ibuprofen after intake of medication. Indeed blood pressure declined over time in all three conditions, probably due to the long resting time.

### Measurement of pain

For the assessment of pain on the visual analogue scale (VAS) no significant difference was found between the three conditions indomethacin, placebo and ibuprofen (mean±SD A= 2.25±1.7; B=2.42±1.9; C=2.37±1.5).

### Adverse events

Before taking the blood sample the participants were asked to report any adverse events during the study. Adverse events were mild to moderate and short-lasting.

## Discussion

In this study we were not able to show an inhibitory effect of indomethacin or ibuprofen on the parasympathetic output of the trigeminal autonomic reflex. This is contrary to previous studies in rodents which suggested that indomethacin may have an effect on the parasympathetic reflex arc. This animal study has shown that neuronal firing on the trigeminal nociceptive brainstem neurons is reduced under indomethacin if the superior salivatory nucleus was also stimulated, and thus the trigeminal autonomic reflex arc was simultaneously activated. In this rodent model indomethacin inhibited evoked firing in the trigeminocervical complex by 30% whereas evoked firing was only inhibited by 15% when nociceptive input came from the dura alone. Naproxen also inhibited neuronal responses but to a lesser extent than indomethacin. In this study indomethacin also reduced lacrimal duct flow in rats, which is correlated to cranial autonomic discharge (25,26). The important point here is that in this model, naproxen had no effect on the parasympathetic outflow to the cranial vasculature. Therefore, the data suggests that both indomethacin and naproxen inhibit neuronal firing in the trigeminal nociceptive nucleus and are therefore “pain killers” but indomethacin also acts specifically on parasympathetic projections from the superior salivatory nucleus and thus further inhibits trigeminal and autonomic responses. This specific effect may explain why indomethacin but not naproxen is unique in its therapeutic effect in PH and HC (26).

One could argue that KOS directly provoked parasympathetic stimulation and that the autonomic response (lacrimation) is not mediated versus the trigemino-autonomic reflex arch. However, since the postganglionic parasympathetic neurons have no specific receptors that could be evoked with the aim to send signals to the effector organ, it needs a central signal to evoke a transmitting signal to the target, i.e. the lacrimal gland here. Stimulating the trigeminal nerve would elicit parasympathetic discharge using the VIIth cranial nerve via the sphenopalatine ganglion (31). In humans, the trigeminal autonomic reflex arc is easily evoked using nasal kinetic oscillation/KOS, allowing the differentiation between nociceptive and mechanic input by eliciting the same parasympathetic response validated by the Schirmer test (32). Using this method in humans our study showed no effect of indomethacin on this reflex, questioning the transferability of the animal data (26) into humans. We again note that the animal study used a direct stimulation of the superior salivatory nucleus and that this stimulation method and also the (in this animal model indirect) method to quantify lacrimation (26) is not directly comparable to our study.

If indomethacin does not affect cranial parasympathetic discharge, how does it elicit its unique therapeutic effect on PH and HC compared with other NSAIDs? A study by Summ and colleagues (24) showed a significant reduction of electrically induced (neurogenic) vasodilation in the cerebral meningeal vessels by ibuprofen and indomethacin. However, NO-induced vasodilation was only reduced by indomethacin and not ibuprofen. The mechanism of NO induced vasodilation is based on an activation of soluble guanylate cyclase by NO which increases the synthesis of cyclic guanosine monophosphate (CGMP). CGMP in turn activates the cyclic GMP-dependent protein kinase (PKG) (33) which phosphorylates ion channels, enzymes and receptors, resulting in a decrease of intracellular Ca^2+^ inducing smooth muscle relaxation in the blood vessels (34). The effect mechanism of indomethacin particularly within that cascade is still not fully understood. It is thought that its unique characteristics might be based on non-prostaglandin effects. A study by Wang and colleagues suggests a direct effect on pH-sensitive mechanisms within the cell (35) that play a role in cerebral blood flow regulation. Furthermore, it has been shown that neither indomethacin nor ibuprofen were able to reduce CGRP induced vasodilation, again suggesting an NO-related effect of indomethacin (24). Goadsby and colleagues have shown that NO might act as a neurotransmitter in the activation of parasympathetic arc of the trigeminal autonomic reflex, with parasympathetic fibers originating in the facial nerve (VII), which leads to cerebral vasodilation in cats (36). It needs to be pointed out, that indomethacin is given as a daily preventive in headaches such as PH and HC, whereas our study used a rather acute setting in healthy volunteers. It is possible that indomethacin acts in a clinical context via another mechanism that induces long term changes to the trigeminal autonomic reflex to mediate its effects.

## Conclusion

The findings of the present study contribute to the understanding of the effect mechanisms of indomethacin in TACs, especially in HC and PH suggesting that indomethacin is not simply effective by specifically modulating the parasympathetic reflex arc. Since we investigated healthy volunteers we cannot make inferences regarding the pathophysiological activation of the trigeminal autonomic reflex, i.e. in patients. Our data suggests however, that indomethacin may be effective through other, for example direct neuronal or NO-dependent inhibitory pathway activity (3).

## Article Highlights

- Unlike other non-steroidal anti-inflammatory drugs, indomethacin has been shown to be highly effective in two forms of trigeminal autonomic cephalalgias. This specificity is unique in the headache field
- Previous findings suggest the involvement of the trigeminal autonomic reflex to play an important role in the pathophysiology of these diseases.
- In our study, neither indomethacin nor ibuprofen had an inhibitory effect on the trigeminal autonomic reflex and blocking this reflex may not be the treatment mechanism of indomethacin
- Indomethacin may be effective through other mechanisms, for example direct neuronal or NO-dependent inhibitory pathway activity
